# New products

**DOI:** 10.1155/S1463924695000319

**Published:** 1995


Journal of Automatic Chemistry, Vol. 17, No. 5 (September–October 1995), pp. 201–204

### New products

### Electronic books

Adept Scientific has announced the Mathcad Authoring Kit for electronic books. Based on Mathcad PLUS 5.0, and compatible with all previous versions and platforms of the software, the Authoring Kit enables organizations to develop Mathcad Electronic Books for the first time. Of particular benefit to commercial and education organizations, the new product offers multiple and company-wide access to standard calculations and technical solutions.

Electronic books for Mathcad have important commercial advantages as they offer organizations fast and effective, yet controlled and secure, access to standard calculation sheets. Important features, such as Hypertext and Word Search, improve productivity by giving users swift access to specific solutions; for example, searching for keywords such as 'modulus' gives fast access to the modulus of elasticity for particular materials.

The books provide live, interactive mathematics which resemble a printed page. While text, graphics, equations and diagrams appear together on a page, they can all be modified, in real-time, by the user. This allows users to monitor and learn as equations, graphs or answers are updated automatically. Because Electronic Books are produced in familiar mathematical notation, users do not need programming skills.

*For further information contact Adept Scientific Micro Systems Ltd, 6 Business Centre West, Avenue One, Letchworth, Herts. SG6 2HB, UK. Tel.: 01462 480055.*

### Solvent recovery system

The B/R 8400, which has now received type certification, is one of a range of fully automatic systems designed to separate two or more solvent fractions from waste streams. Purity of recovered components is to analytical standards, and achieved using a spinning band distillation technique. Commonly used in industrial, medical and laboratory environments, its use reduces solvent purchase and disposal costs by as much as 95%.

The 8400 Model features a programmable microprocessor controller capable of storing up to eight distillation processes and features automatic fraction collection. Extensive monitors and controls regulate all operational parameters, with instant alarms and safety shutdown devices if a failure is ever detected. To comply with the certification, the instrument now includes the fitting of an additional vapour sensor to detect accidental leakage of any solvent within the cabinet and several minor electrical changes.

*For further information on the range of B/R products contact Electrothermal Engineering, 419 Sutton Road, Southend on Sea, Essex SS2 5PH, UK. Tel.: 01702 612211.*

### Autosampler

The Skalar range of automatic wet chemistry analysers has now been extended with a fully random access autosampler. Designed to meet the needs of the medium-sized routine laboratory, the sample station has a capacity of 140 samples in four racks. These comprise 35 samples each, with 11 separated containers for calibration and control standards. Any combination of samples and standards can be run. Overrange samples are automatically diluted and offered for re-analyses to the analyser.

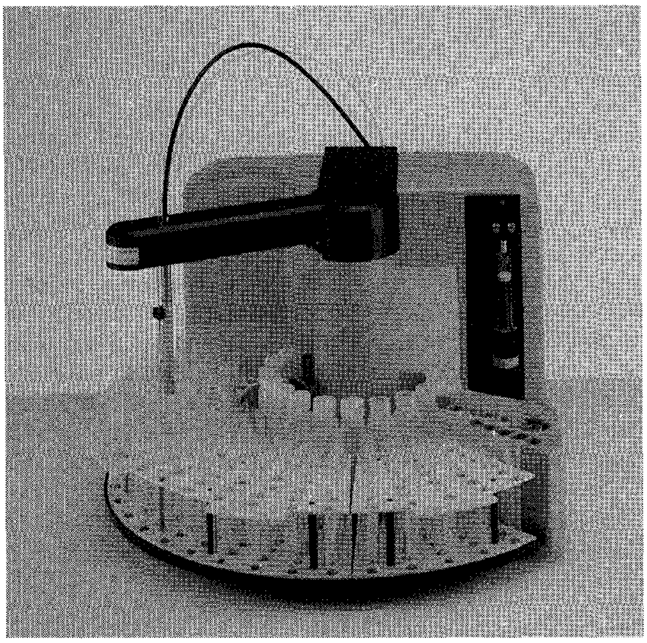

*Intelligent autosampler from Skalar.*

*For more information contact Skalar Analytical B.V., PO Box 3237, 4800 DE Breda, The Netherlands. Tel.: 31 76 225477; fax: 31 76 222195.*

### Titration with a PC

The Metrodata TiNet 2 is a Windows program for titrations which supports routine setup and complex titration systems. The program covers all aspects of titration, such as method generation, sample treatment with live titration curve and processing of the silo memory, the documentation and archiving of the results, post-processing of data and the export of the results.

0142-0453/95 $10.00 © 1995 Taylor & Francis Ltd.

*Methods*

A graphical method editor allows simple and method generation. The individual building blocks of the titration methods, such as titration, dispensing, measurement, calculations and documentation, can be linked with one another in any order and number. And the PC user interface offers new possibilities: individual report formatting or the arithmetic linkage of several determinations in one sample. IF decisions and inquiries or messages to the user can be built into the method at any point.

The methods are stored on the hard disk (30 characters are available per method for the identification of the stored titration methods) and can be printed out at any time. In addition, any type of description or standard operating procedure (SOP) can be stored with each method and can be examined when desired. A backup of the tiration methods on various media (diskette, CD, etc.) is possible at all times. So three important requirements of GLP are fulfilled.

*Titration*

The user can enter his/her name, select the titration method for each sample, and enter the sample designation (three identifications, each with 30 characters) and the sample mass. After the start of the method, the titration curves can be shown live on the PC monitor in various ways (volume/measured value, volume/time, measured value/time, first derivative/volume, etc.), depending on the type of titration. If several titrations are performed in succession in a method, the current titration curve always appears on the screen. With simultaneous titrations, all titration curves appear in parallel on the monitor. For work with relatively large sample series, 'silo' (push-up) memories are available. These can be individually organized, filled with sample data and stored. The incorporation of urgent samples in an ongoing series is always possible. The titrations can be processed in the background of the PC at any time thus allowing it to be used for other tasks in the laboratory.

*Results*

All titration data (parameters, results, titration curves etc.) of a processed sample can be stored. The user has complete freedom of choice when organizing the database (specific to product, customer, time etc.). The database has three windows. One of these offers an overview table of all titrations; a line exists for each determination. The appearance of this table and hence also the contents of the table can be freely configured.

The titration data can be printed out and stored. They can be exported from the TiNet database to data processing programs (for example, Access, Excel, dBase) and to LIMS. As the TiNet database has the same format as Access, the data can be read directly by the two Microsoft programs Access 2.0 and Excel 5.0.

*Further information from Metrohm UK Ltd, Metrohm House, Unit 2, Top Angel, Buckingham Industrial Park, Buckingham MK18 1TH, UK. Tel.: 01280 824824.*

### Water testing

A booklet from Dr Bruno Lange (UK) of Camberley, Surrey, describes the company's continued research and development of new techniques for more efficiency in water testing. The booklet examines such techniques as ready-to-use reagents, simplified dosing systems and test procedures involving reduced amounts of pollutants. The company handles the LASA family of instruments. LASA 10 already contains most of the data needed for everyday waste water analysis in sewage treatment plants, while LASA 20 has the additional ability to determine metal and anion concentrations. LASA 10 also features Dr Lange's specially developed sensor array technology, a brand new optical system enabling the photometer to be operated quickly without filters, thus eliminating any risk of error.

Also described is the CADAS 30 spectrophotometer, which was designed as a fast, reliable and convenient method of water analysis, tailored to the requirements of recurrent routine daily analysis. The CADAS 50, like its parent system, features IBR (Integrated Barcode Reading) technology, enabling fast and precise reading of all Dr Lange cuvette tests, which are each labelled with a special bar code containing all the necessary information for automatic measurements to be performed. Additionally, CADAS 50 features a unique automatic zero facility, thus allowing the zero value of individual tests to be stored and used when the result of each measurement is calculated.

Cuvette tests are the core element of the company's analysis systems, operating not only as a precision measuring vessel but also as a transport container, reaction vessel and disposal unit, with the barcode containing all the test-specific data required for automatic evaluation. The Lange range includes tests for nutrients, chemical oxygen demand (COD), nitrogen, phosphorus and heavy metals.

Other products include the Addista system, used for the quality control of operational analysis combining an external and independent standard, and the new Sensor-BOD, a fast, precise and simple way of measuring the pollution of waste water with biodegradable substances, giving a measured value in two minutes.

*Copies of the booklet from Robin E. Norman, Dr Bruno Lange (UK) Ltd, Argent House, Frimley Road, Camberley, Surrey GU15 2PP, UK. Tel.: 01276 677233/677255; fax: 01276 677307.*

### Nitrogen determination

For over 100 years, scientists in the food, crop production, water, environmental and explosives laboratories have determined nitrogen content by the method developed by Johan Kjeldahl in 1883. In the Kjeldahl method, acid digestion with metallic catalysts converts proteins, aminos and other organic nitrogenous compounds into ammonium compounds, these are then reacted with boiling alkali to liberate ammonia which is distilled into a receiver flask containing boric acid and titrated.

As many aspects of the Kjeldahl method are potentially hazardous to the analyst or the environment, for example use of toxic catalysts and boiling caustic solutions, the Metrohm Application laboratory have developed a new method for nitrogen determination. This new method requires only 0·5 mg copper per sample as a catalyst, needs no boric acid, only uses as much alkali as is needed to neutralize the digestion mixture and makes the distillation stage superfluous. Up to 50 samples can be automatically analysed daily using this method; organic nitrogen is digested using concentrated sulphuric acid and the digested samples are transferred directly to the Metrohm 735 sample changer. The first titration neutralizes excess sulphuric acid with sodium hydroxide. The addition of formalin then converts the ammonium sulphate to hexamethylenetretramine (urotropine):

$$2(NH_4)_2SO_4 + 6HCHO \rightarrow (CH_2)_6N_4 + 2H_2SO_4 + 6H_2O$$

As a side-product of this reaction sulphuric acid is generated which is titrated with sodium hydroxide with the excess formaldehyde being oxidized with hydrogen peroxide to formic acid. The whole analysis, including disposal of the sample and rinsing of the measuring assembly at the end of the determination, takes about 10 min.

The Metrohm systems for nitrogen determination comprises of a 670 Titro processor, three Dosimats for titrant and reagent addition plus the new 735 sample changer.

*Details from Metrohm UK Ltd, Metrohm House, Unit 2, Top Angel, Buckingham Industrial Park, Buckingham. MK18 1TH, UK. Tel.: 01280 824824.*

### Hazardous area weighing systems

Avery Berkel has developed a range of instruments with full UK government approvals for trade weighing in environments classified as hazardous. The 'Loadstar Ex' range includes three intrinsically safe indicator systems, which can be used with scale platforms or vessels in hazardous areas. They meet the needs of virtually every application involving explosion hazards, volatile materials or combustible dusts encountered in pharmaceutical manufacture, flour milling, refineries, paint production, chemical, petrochemical and process industries.

Each model offers different levels of functionality and data capture—from the L117Ex weigh-only digital indicator, to the sophisticated L227Ex multi-purpose indicator system with on-board software to control the entire spectrum of weighing-related factory operations.

Each model stores and recalls tare values. External control of valves, feeders and other devices can be carried out by 'Loadstar Ex', while a 'trips' option permits interface to automatic filling equipment. An in-flight compensator automatically adjusts target weights to control the consistency of batching and guard against costly product give-away.

For standard applications, the complete weighing installation (platform, indicator and power supply—which can be mains or battery) is located in the hazardous area where it is needed. Communication within and outside the hazardous area is achieved by fire optic transmission and all 'Loadstar Ex' models are compatible with a wide range of industry-standard printers, PLCs and computers for comprehensive data collection, and even full remote process control. Data output is programmable and users may select the exact order and format of management reporting they require.

*For more information contact Avery Berkel UK, Sertec House, West Bromwich Road, Tame Bridge, Walsall WS5 4BD, UK. Tel.: 01922 434343.*

### Gas chromatography solutions

### HP INTRODUCES THE HP 6890 SERIES GC SYSTEM FOR THE NEW STANDARD OF HIGH-PERFORMANCE GAS CHROMATOGRAPHY

Hewlett-Packard Company launched an integrated gas chromatography (GC) system at Pittcon 95, which gives chromatographers a new level of performance and push-button control, and easy regulatory compliance. The new GC system, the HP 6890 series, includes a new automatic liquid-sampler system (ALS) with increased throughput and automation, a choice of an HP ChemStation data handler/controller for GC and ALS control, and an integrator that supports the GC features completely. The system was designed to deliver optimal performance when coupled with HP sample preparation, sample introduction and data handling products. The HP 6890 series GC offers a smooth transition for users of HP 5890 series gas chromatographs through methods compatibility.

*Electronic pneumatics control*

The HP 6890 series GC features electronic pneumatics control (EPC), which provides complete electronic control of all gas pressures and flows. Onboard sensors automatically compensate for ambient temperature changes and pressure differences. By providing stable results, EPC reduces recalibration frequency and improves laboratory productivity. It also decreases system operating costs through gas savings and faster set-up time, and reduces equilibration time after changing set-points.

The HP 6890 series GC pneumatics optimize performance of the split/splitless inlet. Its forward pressure control in splitless injection mode significantly reduces the risk of sample loss and maximizes accuracy and reproducibility. In contrast, mass flow control coupled with back pressure control in split injection mode maximizes reproducibility and accuracy, and allows electronic adjustment of split ratios. The net result is that sample-introduction conditions are optimized individually for the two most popular injection techniques. In addition, all injection parameters are recorded in the method.

Ease of use features include:

(1) All pneumatic parameters, including split ratios, can be set from the keyboard to eliminate routine use of the bubble meter.
(2) An expanded colour-coded keyboard gives analysts direct access to control functions without the need for shift keys.
(3) A five-method storage capability for all GC set-points reduces set-up time and increases reproducibility.

(4) An information key defines all set-points and advises analysts on appropriate input ranges to make methods set-up fast and easy.
(5) A four-line alphanumeric display guides analysts through methods set-up with step-by-step prompting for set-point parameters.
(6) Built-in HPIB and RS-232 communication ports provide easy system interconnection and allow the GC system to operate in diverse laboratory environments.

*Productivity*

The HP 6890 series GC also offers a range of automation features including: clock-time programming loads and runs a method for complete unattended operation; pre-run programming enters conditions and prepares the system for next injection; gas saver reduces split flow rate after the start of a run and between runs to conserve expensive carrier gas; and post-run programming starts after the chromatographic run, stops data acquisition and sets pressures, flows and temperatures to clean out the system.

Built-in diagnostic capabilities—such as a run log for status information, and modular pneumatic manifolds, inlets and detectors—reduce system maintenance and improve operation.

Capabilities of the new HP ChemStation include a full-featured standard reporting package and a custom report designer for analysts with special reporting requirements. An optional database software package provides standard cross-sample and study reports, as well as trend analysis. The HP ChemStation also improves data transfer to other software, and networking acts as the analyst's gateway into a corporate computing system.

A new integrator offers full compatibility and support of the new HP 6890 series GC features, and includes gas-saver mode, split valve control and post-run programming. It also contains two new industry-standard Personal Computer Memory Card International Association (PCMCIA) slots to accommodate long-term data storage and prints the run deviation log after each analysis.

*Regulatory compliance*

All GC parameters, including gas control, are recorded automatically for every run to improve adherence to Good Laboratory Practice (GLP) and ease regulatory compliance. A run-time log records all changes in set-points during a run.

The HP ChemStation also provides password access to protect against accidental data loss or methods changes. The HP ChemStation controls and monitors all GC parameters and maintains a logbook of all system events that occur while the GC system is running. System-suitability software, which allows analysts to select from a wide variety of chromatographic parameters to monitor and verify system performance, is also available.

For analysts in the pharmaceutical industry or other regulated industries, data and methods are stored together in a binary, read-only file to ensure adherence to regulatory requirements. Software for the HP ChemStation is developed according to International Standards Organization (ISO) requirements and is shipped with a validation certificate. HP also provides tools to give analysts the ability to revalidate the software and GC.

*Further information from Hewlett-Packard Company (102) Analytical Products Group, 1031, PO Box 9000, San Fernando, CA 91341-9981, USA.*

### Screening for metabolic disorders

'*Rapid Screening For Metabolic Disorders From Blood Spots*' is the title of a new application note from Fisons Instruments/VG Organic. A small number of new-born babies may inherit parental genes resulting in metabolic disorders. As in the case of eye colour or height which cannot be permanently changed, many of these diseases are not 'curable' owing to their genetic origin. However, a combination of early diagnosis and establishment of a maintenance course of treatment could result in the significant reduction—and even elimination—of the debilitating effects of many metabolic disorders. Clinically, screening for such inherited disorders should take place as soon as possible after birth, to prevent irreversible damage being caused by the progression of a disorder.

The presence of a metabolic disorder results in an accumulation of abnormal metabolites in the patient. Since 1965, new-born screening programs have been in use, for example testing for phenylketonuria (PKU). PKU is a disorder resulting from phenylalanine hydroxylase deficiency leading to elevated levels of phenylalanine in blood and urine. Sample collection currently involves transferring drops of blood from a heel-prick onto a blotting paper known as Guthrie card, and whilst this particular sample is used in routine PKU screening and a few other tests, it also contains many more metabolites not currently screened routinely. Using mass spectrometry, it is possible to detect these metabolites and use them as indicators of a significant number of metabolic disorders, in addition to PKU.

*Copies from IAS, Queens Avenue, Macclesfield, Cheshire SK10 2BN, UK. Tel.: 01625 434343; fax: 01625 434335.*

